# MAGNETO: An Automated Workflow for Genome-Resolved Metagenomics

**DOI:** 10.1128/msystems.00432-22

**Published:** 2022-06-15

**Authors:** Benjamin Churcheward, Maxime Millet, Audrey Bihouée, Guillaume Fertin, Samuel Chaffron

**Affiliations:** a Nantes Université, École Centrale Nantes, CNRS, LS2N, UMR 6004, Nantes, France; b Nantes Université, CNRS, INSERM, l’institut du thorax, F-44000 Nantes, France; c Nantes Université, CHU Nantes, SFR Bonamy, F-44000 Nantes, France; d Research Federation for the study of Global Ocean Systems Ecology and Evolution, FR2022/Tara Oceans, Paris, France; University of California San Diego

**Keywords:** computational workflow, metagenome-assembled genomes, metagenomics, microbiomes

## Abstract

Metagenome-assembled genomes (MAGs) represent individual genomes recovered from metagenomic data. MAGs are extremely useful to analyze uncultured microbial genomic diversity, as well as to characterize associated functional and metabolic potential in natural environments. Recent computational developments have considerably improved MAG reconstruction but also emphasized several limitations, such as the nonbinning of sequence regions with repetitions or distinct nucleotidic composition. Different assembly and binning strategies are often used; however, it still remains unclear which assembly strategy, in combination with which binning approach, offers the best performance for MAG recovery. Several workflows have been proposed in order to reconstruct MAGs, but users are usually limited to single-metagenome assembly or need to manually define sets of metagenomes to coassemble prior to genome binning. Here, we present MAGNETO, an automated workflow dedicated to MAG reconstruction, which includes a fully-automated coassembly step informed by optimal clustering of metagenomic distances, and implements complementary genome binning strategies, for improving MAG recovery. MAGNETO is implemented as a Snakemake workflow and is available at: https://gitlab.univ-nantes.fr/bird_pipeline_registry/magneto.

**IMPORTANCE** Genome-resolved metagenomics has led to the discovery of previously untapped biodiversity within the microbial world. As the development of computational methods for the recovery of genomes from metagenomes continues, existing strategies need to be evaluated and compared to eventually lead to standardized computational workflows. In this study, we compared commonly used assembly and binning strategies and assessed their performance using both simulated and real metagenomic data sets. We propose a novel approach to automate coassembly, avoiding the requirement for *a priori* knowledge to combine metagenomic information. The comparison against a previous coassembly approach demonstrates a strong impact of this step on genome binning results, but also the benefits of informing coassembly for improving the quality of recovered genomes. MAGNETO integrates complementary assembly-binning strategies to optimize genome reconstruction and provides a complete reads-to-genomes workflow for the growing microbiome research community.

## INTRODUCTION

Genomes are a valuable resource for characterizing and understanding the diversity, ecology, and evolution of microbial organisms in the laboratory as well as in natural environments. As culture-based approaches have been historically used to recover genomes and enrich reference databases, current knowledge from most reference bacterial genomes comes from axenic cultures. However, despite the improvement of culture-based approaches to cultivate novel microorganisms, the number of organisms that can be isolated and cultivated remains mainly constrained by specific growth conditions. Depending on the considered environment, it is estimated that a proportion of only 0.1% to 1% of all microbial genomes could be cultivated (1, 2).

The rise of metagenomic studies, thanks to the rapid development of high-throughput shotgun sequencing, has allowed direct access to the diversity and functional potential of naturally occurring microorganisms, bypassing the cultivation bottleneck. For more than a decade, various studies have reconstructed genomes from metagenomes and contributed to describing thousands of novel microbial clades belonging to diverse environments, such as the human gut (3), soils, and aquatic environments (4, 5).

The reconstruction of these draft genomes, commonly called metagenome-assembled genomes (MAGs), has now become a common approach, with much software developed during the last decade (6–9). As for the reconstruction of genomes from single organisms, MAG reconstruction can be split into two main steps: first, the *assembly* of the reads obtained from the sequencing into longer sequences called *contigs*; second, the *binning* of these contigs into MAGs, mainly using their compositional and/or abundance similarities. However, MAG reconstruction can face several limitations including gaps, sequencing errors, local assembly errors, contigs chimeras, and bin contamination (i.e., the inclusion of contigs belonging to different genomes in the same bin). The binning of contigs may also miss genomic regions in which nucleotidic composition differs significantly from the genome average, such as ribosomic RNA regions, or mobile elements (10). These limitations can be partially addressed by several quality checkpoints, misassemblies detection, and manual curation (11).

In addition, low abundance organisms are usually harder to recover, due to limited reads information during the assembly process (12). When shallower sequencing is performed (i.e., the predefined number of bases the sequencer outputs is low), reads from low-abundant genomes will be rare, and thus their assembly into contigs will be more difficult, as assemblers tend to consider these reads as erroneous and discard them. A common approach to increase the abundance of rare reads is to adapt the assembly strategy, that is, not assembling a unique metagenomic sample (single assembly), but *coassembling* several samples together. Coassembly will then tend to increase the number of occurrences of rare reads, and consequently incorporate them into resulting contigs, thereby capturing a higher fraction of the diversity within the samples. Coassembly strategies have been instrumental for recovering higher numbers of MAGs (13, 14); however, this approach increases the probability of generating fragmented assemblies (12, 15).

The genome binning process consists of classifying contigs usually based on similarities of their sequence composition, their abundance, or their taxonomic affiliation. In most existing softwares, binning is performed using two main metrics, namely, sequence composition (6) and contigs abundance (7). Sequence composition is defined as the frequencies of all tetra nucleotides within the contig sequence, called TNF (for tetranucleotide frequency). The abundance (or coabundance) represents the mean vertical coverage of the contig in one (or several) sample(s). Other metrics, such as taxonomic affiliation of the contigs, may also be used to determine which contigs belong to the same bin (16). The principal differences between existing binning software usually involve the algorithm used to group contigs into genome bins. Most successful softwares have used density-based clustering (17), Gaussian mixture models (7), affinity propagation (18), or graph clustering (9). Other methods can also perform binning on genes rather than contigs, relying on the presence of coabundant genes within metagenomes, such as canopy clustering (19) and MSPminer (20). The objects reconstructed by these methods may not be qualified as MAGs, and are commonly referred to as metagenomic species (MGS) and coabundance groups (CAGS).

Extracting knowledge from raw metagenomics data requires handling several specific tasks, from assembly to gene calling and annotation, each of them often performed using dedicated software. Today, dedicated workflows for these tasks start to emerge (21), but they are still not widely adopted by the community. Users commonly face choosing, configuring, and running different tools, which can be challenging and time-consuming. Recently, several metagenomics workflows have been developed (22–25), often using specific default parameters for each integrated software. However, these workflows usually suffer from limits toward either the assembly step or the genome binning step. In workflows allowing coassembly, sets of samples to coassemble have to be determined and manually specified by the user, implying some *a priori* knowledge about the microbial ecosystem under study. Besides, only a single workflow (25) allows the user to compute coabundances from metagenomes that have not been included in the assembly. As computing coabundance profiles of contigs from multiple metagenomes may increase the precision of the metric (9, 26), the impossibility of computing large-scale coabundance may be considered a limitation of these workflows.

Here, we present MAGNETO, a fully automated workflow for genome-resolved metagenomics, implementing a coassembly module that integrates a nonsupervised method to define sets of samples to coassemble without *a priori* knowledge. It also includes complementary strategies to compute abundance metrics from one to *n* metagenomes, even if they do not participate in the assembly process. In this study, we tested our coassembly module on a set of marine metagenomes, against a coassembly relying on existing knowledge. We also benchmarked four different assembly-binning strategies for MAG reconstruction, on diverse data sets ranging in complexity from a mock data set representing a small bacterial community to human gut microbiome communities.

## RESULTS

### Determining coassemblies using metagenomic distances.

In reference 13, the authors studied the abundance of diazotrophic bacteria in oceanic surface metagenomes and showed that nitrogen fixation is an important feature of the prokaryotic communities living on the ocean surface. As microbial genetic distances often covary with geographic distances in several habitats (27), coassemblies were performed based on the geographic coordinates of the metagenomes (i.e., metagenomes belonging to the same oceanic region were coassembled). In the euphotic zone, an average higher microbial community similarity within than across ocean regions has been observed at a global scale, although a separation by regional origin is unclear (28), as other environmental factors (e.g., ocean currents) can modulate genetic proximity between populations (29). In consequence, two metagenomes geographically close do not necessarily share the highest proportion of genomes, and two metagenomes belonging to the same ocean region may not be closer to metagenomes from other regions.

**FIG 1:**
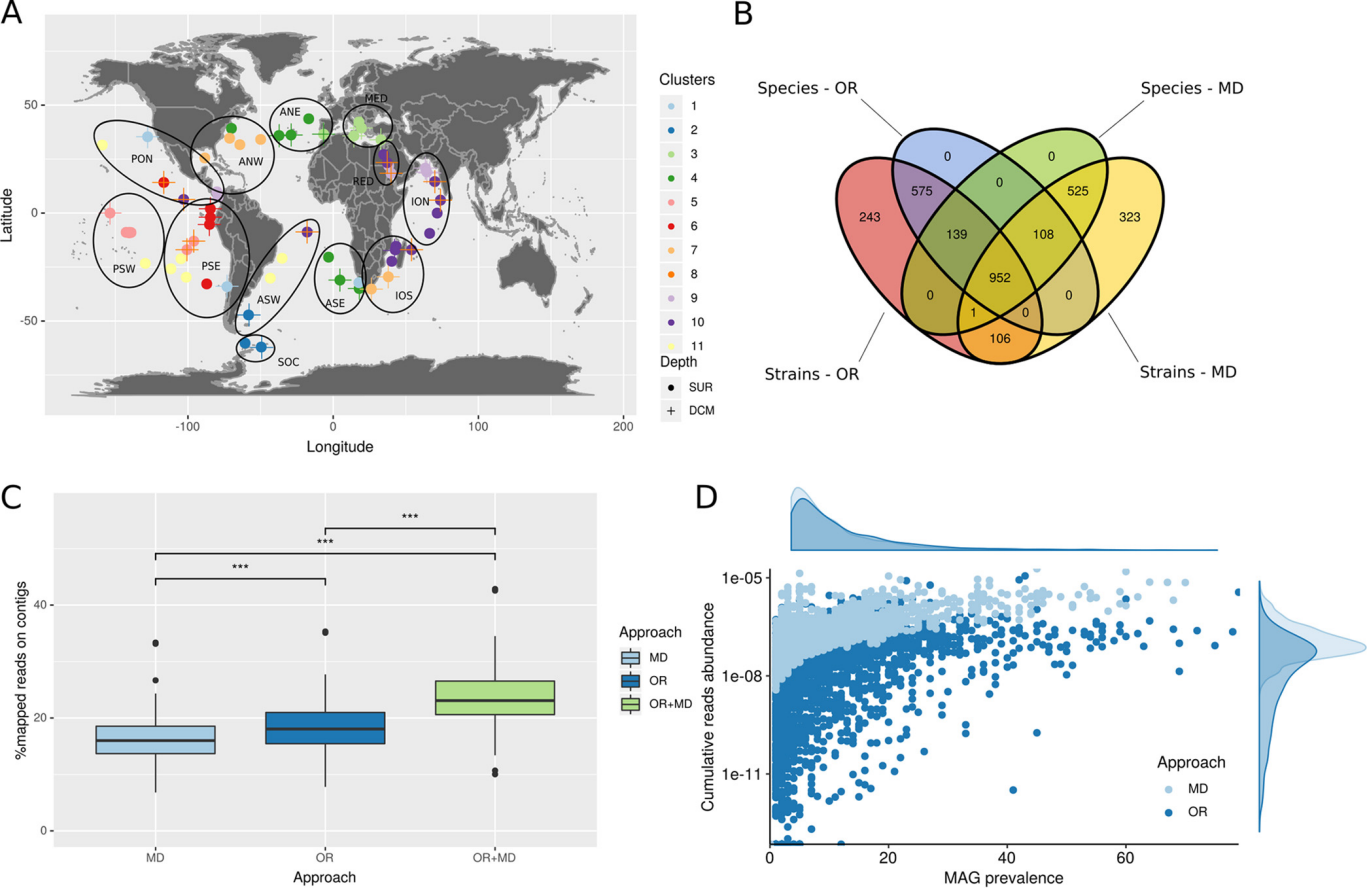
Evaluating the metagenomic distance-based (MD) approach against the oceanic regions (OR) approach for delineating groups of samples to coassemble. (A) Repartition of clusters obtained with Simka. Each dot represents a metagenome obtained at a sampling station, with metagenomes located at surface (SUR) represented as dots, and metagenomes situated at the deep chlorophyll maxima (DCM) depth as crosses. Colors represent the cluster to which the metagenome belongs. Oceanic regions are represented as dark circles: ANE, Atlantic North-East; ANW, Atlantic North-West; ASE, Atlantic South-East; ASW, Atlantic South-West; ION, Indian Ocean North; IOS, Indian Ocean South; MED, Mediterranean Sea; PON, Pacific Ocean North; PSE, Pacific South-East; PSW, Pacific South-West; RED, Red Sea; SOC, Southern Ocean. (B) Repartition of the common MAGs obtained after common dereplication between the two approaches. (C) Percentage of mapped reads on MAGs reconstructed by each approach, and on combined MAGs from both approaches, considering all mapping reads. MD, metagenomic distance; OR, oceanic region; OR+MD, MAGs from OR and MD approaches were combined together prior to reads mapping. (D) Prevalence-abundance plot for MAGs reconstructed by both approaches (*x* axis: MAG prevalence = number of metagenomic samples in which a MAG has a horizontal coverage above 0.3; *y* axis: MAG cumulative abundance = percentage of mapped reads divided by the length of the MAG).

Given that the main goal of coassembly is to increase the proportion of reads belonging to a given strain or species, we propose to identify sets of samples to coassemble using metagenomic distances. To the best of our knowledge, very few studies have used sequence-based compositional distances to guide metagenomic coassembly. Historically, metagenomic compositional distances have mainly been used to compare metagenomic samples (30) or MAGs (14), but not to actually guide the coassembly process. However, a few recent studies have started to use metagenomic-based distances combined with clustering to guide the coassembly process of metagenomes (31–33), while another study has used metagenomic distances to guide the cobinning (or comapping) process (34). Here, we computed distances between metagenomes using Simka (35), and identified optimal clustering solutions using the Silhouette index (36) to delineate unsupervised sets of samples to coassemble. Applying this approach on the same set of metagenomes (*n* = 93), as in reference 13, we identified 24 optimal clusters. This number of clusters is significantly higher compared to the 12 clusters (Fig. 1A) based on oceanic regions, which suggests that a different partition may be more relevant for coassembly. As this optimal clustering generates smaller clusters, in order to ensure a fair comparison between both approaches, we further identified a suboptimal clustering (Fig. S1 in the supplemental material) whose number of coassembly sets is comparable to the number of oceanic regions used in reference 13. This second clustering identified 11 clusters, which did not match the oceanic regions previously defined (Fig. 1A).

10.1128/msystems.00432-22.1 FIG S1 Identifying metagenomic distance-based optimal clusters for *TARA* oceans metagenomes Silhouette scores obtained when clustering the metagenomes into *k* groups. *x* axis: values of *k* tested, *k* varying from 2 to *n*–1, with *n *= 93 (the total number of samples used). *y* axis: values of the Silhouette score estimated for value *k* tested. A high Silhouette score yields a better clustering. A maximum score is observed for *k *= 24, which thus represents the optimal clustering. Download FIG S1, PNG file, 0.1 MB. Copyright © 2022 Churcheward et al. 2022 Churcheward et al. https://creativecommons.org/licenses/by/4.0/ This content is distributed under the terms of the Creative Commons Attribution 4.0 International license.

To evaluate the potential impact of coassembly on assembly quality, we computed classical assembly quality metrics (N50 and L50) for both approaches. The metagenomic distance (MD)-based and the oceanic regions (OR) approaches actually reconstructed contigs of similar quality. No significant differences were detected in either the number of misassemblies or the N50 and L50 metrics (Fig. S2). When considering the total number of bins generated following both coassembly strategies, we found that both approaches reconstructed very similar numbers of bins: 10,748 bins generated using the MD approach, and 10,233 bins using the OR approach (Fig. 1B). To further compare both coassembly strategies, as these bins may be very different in composition, we performed MAGs dereplication (37). The MD approach systematically reconstructed more MAGs than the OR approach, at both species (95% average nucleotide identity [ANI]) and strain (99% ANI) levels (Fig. S3B). Considering MAGs quality, medium quality (MQ) MAGs reconstructed by the MD approach were significantly more complete (Mann-Whitney U test, *P* = 0.01; Fig. S4B), but evaluated as more contaminated (using checkM) than MQ MAGs reconstructed with the OR approach (Mann-Whitney U test, *P* = 6.828·10^−05^; fig. S4D). However, when considering the GUNC contamination metric (38), contamination levels observed in MQ MAGs of the MD approach were significantly lower than MQ MAGs of the OR approach (Mann-Whitney U test, *P* = 2.352·10^−07^; Fig. S4F). Because GUNC assesses gene contamination based on all taxonomically annotated genes in a given genome, this latter approach may be considered as more robust than the checkM metric, and lead us to conclude that the MD approach actually reconstructed less contaminated MAGs. We found no significant differences in quality (completeness and contamination) for high quality (HQ) MAGs reconstructed by both approaches (Fig. S4). In addition, taxonomic annotations of strain-level dereplicated MAGs revealed a higher diversity recovered in the MD MAGs compared to the OR MAGs (Fig. S5) in terms of number of distinct bacterial taxa, with a greater number of annotated MAGs in MD (*n* = 2,006) compared to OR (*n* = 1,869) MAGs.

10.1128/msystems.00432-22.2 FIG S2 Quality of the assembly of each TARA approach. (A) Comparison of the number of misassemblies normalized by the number of contigs per assembly of the metagenomic distance (MD) and the oceanic regions (OR) approaches. (B) Number of mismatches detected per 100kb of alignments in contigs per approach. (C) N50 values: N50 represents the shortest contig length to cover at least 50% of the metagenome assembly. (D) L50 values: L50 represents the smallest number of contigs whose added lengths cover 50% of the metagenome assembly. No significant differences were found between the two approaches (Mann-Whitney U test). Download FIG S2, PNG file, 0.1 MB. Copyright © 2022 Churcheward et al. 2022 Churcheward et al. https://creativecommons.org/licenses/by/4.0/ This content is distributed under the terms of the Creative Commons Attribution 4.0 International license.

10.1128/msystems.00432-22.3 FIG S3 Evaluating the metagenomic distance-based (MD) approach against the oceanic regions (OR) approach for delineating groups of samples to coassemble. (A) Total number of bins obtained after binning step. HQ, high quality; MQ, medium quality; LQ = low quality. (B) Number of reconstructed MAGs after independent dereplication for each approach. OR, oceanic region; MD, metagenomic distance. Species resolution consists of a 95% ANI score dereplication, while strains resolution consists of a 99% ANI score dereplication. Download FIG S3, PNG file, 0.04 MB. Copyright © 2022 Churcheward et al. 2022 Churcheward et al. https://creativecommons.org/licenses/by/4.0/ This content is distributed under the terms of the Creative Commons Attribution 4.0 International license.

10.1128/msystems.00432-22.4 FIG S4 Quality of reconstructed MAGs from the marine metagenomes. Comparison of the oceanic region (OR) approach against the metagenomic distance (MD) approach. HQ, high quality; MQ, medium quality. (A) Completeness estimated for HQ MAGs; (B) completeness estimated for MQ MAGs; (C) contamination measured using SCGs for HQ MAGs; (D) contamination measured using SCGs for MQ MAGs; (E) contamination measured using all genes detected in the sequences of HQ MAGs; (F) contamination measured using all genes detected in the sequences of MQ MAGs. Download FIG S4, PNG file, 0.1 MB. Copyright © 2022 Churcheward et al. 2022 Churcheward et al. https://creativecommons.org/licenses/by/4.0/ This content is distributed under the terms of the Creative Commons Attribution 4.0 International license.

10.1128/msystems.00432-22.5 FIG S5 Comparison of the number of taxa retrieved from MAGs reconstructed following each approach. (A) Number of bacterial taxa assigned to MAGs from both approaches, per taxonomic level. (B) Number of archaeal taxa assigned to MAGs from both approaches. Taxonomic annotation was performed using gtdb-tk on dereplicated MAGs at strain level (99% ANI score) using dRep. For each panel, the *y* axis represents the number of taxa found withing the complete set of MAGs of each approach. MD, metagenomic distance (estimated using Simka); OR, oceanic region (from reference 13). Number of MAGs assigned to a bacterial annotation is 1,729 for MD and 1,595 for OR, while there are 276 and 273 MAGs with an archaeal assignation in MD and OR, respectively. Download FIG S5, PNG file, 0.1 MB. Copyright © 2022 Churcheward et al. 2022 Churcheward et al. https://creativecommons.org/licenses/by/4.0/ This content is distributed under the terms of the Creative Commons Attribution 4.0 International license.

Next, we also performed a global dereplication of MAGs in order to compare sets of MAGs recovered by both approaches at species and strain levels (see supplementary Methods). Remarkably, we observed that both approaches reconstructed a very high number of exclusive MAGs (Fig. 1B). The OR approach reconstructed 575 species-level and 243 strain-level MAGs that were not recovered by the MD approach, while the latter did reconstruct 525 species-level and 323 strain-level MAGs that were not recovered by the OR approach. This result strongly emphasizes the influence of the coassembly step prior to genome binning, in particular regarding how metagenomes are grouped for coassembly. Given this observation, we aimed at determining which approach could captured a greater proportion of metagenomic diversity by back-mapping reads on MAGs generated by both approaches. While we observed a lower proportion of reads mapping to MD MAGs, compared to OR MAGs, this proportion significantly increased when mapping on combined MAGs from both approaches. This result confirms that distinct and complementary MAGs are reconstructed using each approach. However, when only considering reads mapping to MAGs detected in samples, (i.e., in which a given MAG has a minimum horizontal coverage [or breadth] of 30%), the MD approach recruited significantly more metagenomic reads compared to the OR approach (Mann-Whitney U test, *P* < 2.2·10^−16^, Fig. 1D). Thus, although the OR MAGs were detected in more samples compared to MD MAGs (Mann-Whitney U test, *P* = 4.6·10^−10^), the MD MAGs significantly improved the number and quality of reconstructed MAGs.

### Benchmarking assembly-binning strategies on simulated metagenomes.

Different strategies for assembly and binning are currently used in the literature, each of them having its own advantages and disadvantages (14). Thus, we defined four assembly-binning strategies representing the most currently used approaches to reconstruct MAGs. Namely, we considered single-assembly (SA; i.e., the assembly of a single metagenome) and coassembly (CA; i.e., the joint assembly of *n* metagenomes) approaches, as well as single-binning (SB; i.e., genome binning solely using [co-]abundance information from metagenome[s] used to perform the [co-] assembly) and cobinning (CB; i.e., genome binning using coabundance information from all metagenomes) approaches. We thus evaluated the following four strategies: single-assembly with single-binning (SASB), single-assembly with cobinning (SACB), coassembly with single-binning (CASB), and coassembly with cobinning (CACB). We compared the performances of these four strategies on three different data sets, the CAMI (15) high-complexity data set, a lower complexity mock data set generated using CAMISIM (39), and a human microbiome data set from the Human Microbiome Project (40).

**FIG 2:**
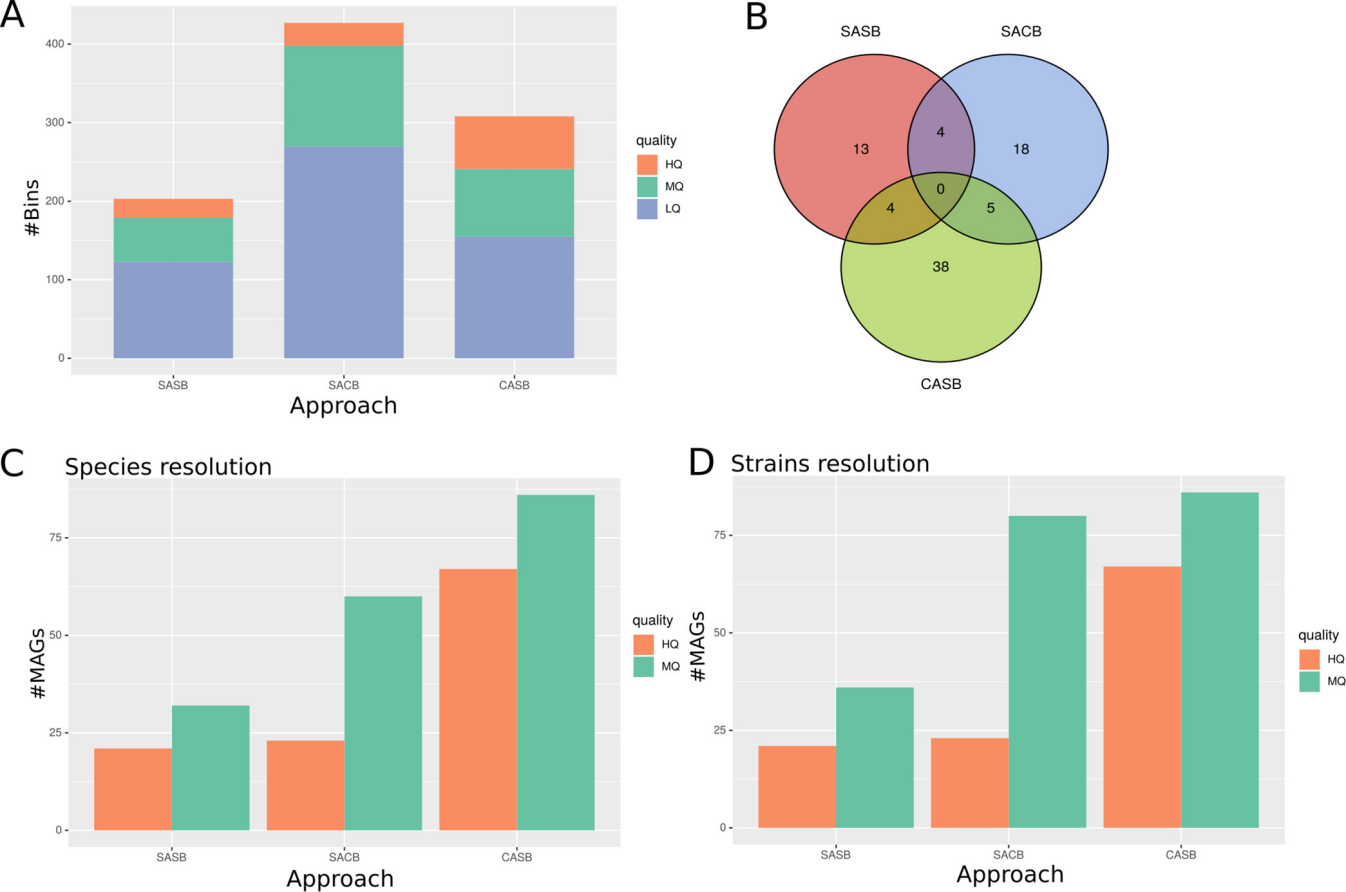
Evaluating assembly-binning strategies on the CAMI data set. (A) Total number of bins obtained after binning step. Colors represent quality of genome bins estimated using CheckM: high quality (HQ), medium quality (MQ), and low quality (LQ). (B) Number of MAGs mapping to a source genome within each strategy, corresponding to the number of expecting genomes in the set of MAGs of each strategy. The diagram thus represents the common genomes found in each strategy. (C, D) Number of reconstructed MAGs after independent dereplication using dRep for each binning strategy, at (C) species resolution, consisting of a 95% ANI score dereplication; and (D) strains resolution, consisting of a 99% ANI score dereplication. SASB, single-assembly single binning; SACB, single-assembly cobinning; CASB, coassembly single binning.

First, to evaluate and compare these four strategies on simulated metagenomes, we applied our MD clustering algorithm on the CAMI high-complexity data set (15). The CAMI high-complexity data set is composed of five metagenomic samples simulated from a community of 596 known reference genomes and 478 circular elements. The optimal solution identified for the coassembly regrouped all five metagenomes, probably due to the small number of metagenomes (*n* = 5) and the fact that they were simulated from the same pool of reference genomes. Therefore, only one coassembly (of all 5 samples) was performed, and the CACB and CASB strategies were thus equivalent. Following genome binning using MetaBAT2 (9), the SACB strategy reconstructed the highest number of bins (>400 genome bins), while the CASB and SASB strategies reconstructed about 300 and 200 bins, respectively (Fig. 2A).

After dereplication, we compared the MAGs obtained for each strategy to the CAMI reference source genomes. When considering the distribution of expected genomes across all three strategies, we observed that the CASB strategy reconstructed more expected genomes than both single-assembly strategies (SASB and SACB). Surprisingly, we did not find expected genomes common to all strategies (Fig. 2B), which highlights the actual complementarity of these strategies. When considering only dereplicated genomes, CASB produced the highest number of MAGs. This difference was clear for HQ MAGs, for which CASB produced about 2.5 times more MAGs compared to single-assembled strategies, with both SACB and SASB generating a comparable number of HQ MAGs (Fig. 2CD).

The number of reconstructed MAGs was also dependent of the dereplication level. At strain level, both single-assembly approaches reconstructed more nonredundant MAGs compared to species level, while CASB reconstructed the same number of MAGs at both species and strain levels. However, this increase concerned only the MQ MAGs, as the number of HQ MAGs remained unchanged (Fig. 2D). We did not find any significant differences in MAG completeness between the different strategies, considering either HQ MAGs or MQ MAGs (Fig. S6A and B). However, we did observe differences in contamination estimated from single-copy genes (SCGs) using checkM. CASB HQ MAGs were less contaminated than SASB (Mann-Whitney U test, *P* = 0.01) and SACB (Mann-Whitney U test, *P* = 0.04) HQ MAGs, while SACB MQ MAGs were less contaminated than SASB MQ MAGs (Mann-Whitney U test, *P* = 0.03) (Fig. S6C and D). When considering MAG contamination estimated using taxonomically annotated genes using GUNC, CASB MAGs were predicted most contaminated (Fig. S6E and F), with significant differences observed with both SASB (Mann-Whitney U test, *P* = 3·10^−4^) and SACB (Mann-Whitney U test, *P* = 2·10^−4^) MAGs. We did not find any other differences in contamination levels between the four strategies.

10.1128/msystems.00432-22.6 FIG S6 Comparison of the quality of MAGs reconstructed with each strategy on the CAMI dataset. HQ, high quality; MQ, medium quality. (A) Completeness estimated for HQ MAGs; (B) completeness estimated for MQ MAGs; (C) contamination measured using SCGs for HQ MAGs; (D) contamination measured using SCGs for MQ MAGs; (E) contamination measured using all genes detected in the sequences of HQ MAGs; (F) contamination measured using all genes detected in the sequences of MQ MAGs. Download FIG S6, PNG file, 0.1 MB. Copyright © 2022 Churcheward et al. 2022 Churcheward et al. https://creativecommons.org/licenses/by/4.0/ This content is distributed under the terms of the Creative Commons Attribution 4.0 International license.

**FIG 3:**
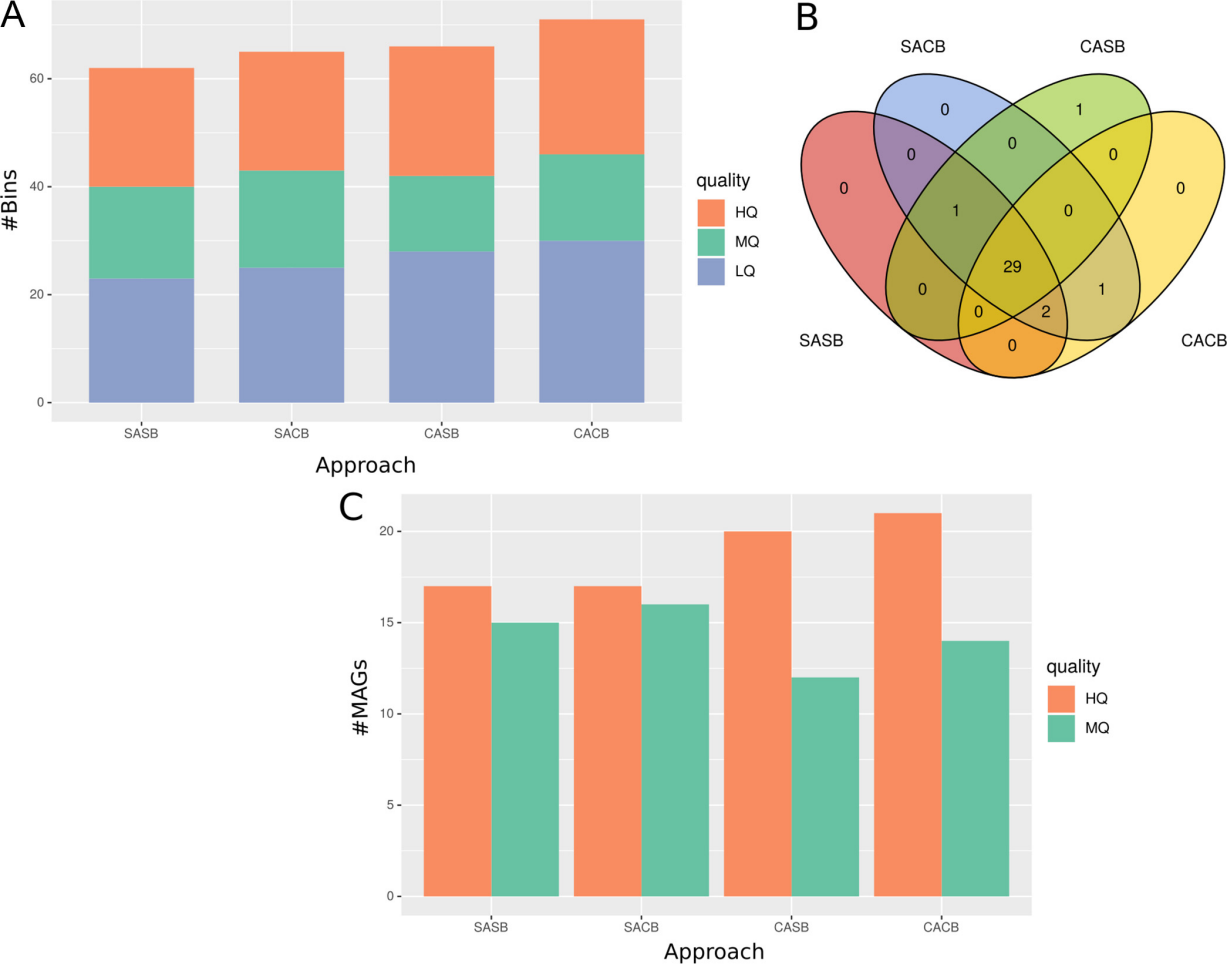
Evaluating assembly-binning strategies on simulated metagenomes. (A) Total number of bins obtained after the binning step. colors represent quality of genome bins estimated using CheckM: high quality (HQ), medium quality (MQ), and low quality (LQ). (B) Number of source genomes found in each strategy. Each number represents the number of times a MAG from a strategy maps against a source genome. Intersections represent common genomes between strategies. (C) Number of dereplicated MAGs obtained, after independent dereplication by dRep for each strategy. As the genomes are all represented with one single strain, dereplication at either species or strain resolution gives the same number of dereplicated genomes, so only one dereplication resolution is shown.

Given that the MD clustering approach did not identify optimal clusters to coassemble within the CAMI data set, we used CAMISIM (39) to simulate an additional metagenomic data set with a higher number of samples and a lower complexity. We thus simulated 20 metagenomes with a similar diversity of 100 reference genomes. On this simulated data set, the MD clustering approach identified 8 optimal clusters to coassemble. Here, the coassembly-based strategies (CACB and CASB) reconstructed more bins than the single-assembly-based strategies (Fig. 3A), also when considering only HQ bins. After dereplication, we aimed to identify expected genomes among recovered MAGs by mapping them to reference genomes used for the metagenome simulation. The majority (*n* = 29) of expected genomes we identified were reconstructed in all four strategies (Fig. 3B). The SACB strategy recovered a short majority of expected genomes (*n* = 33), compared to CACB and SASB (*n* = 32), and CASB (*n* = 31). However, the number of dereplicated MAGs was higher for both coassembly strategies compared to single-assembly strategies (Fig. 3C).

The drop in dereplicated MAGs from single-assembly strategies is likely a consequence of the higher number of assemblies performed in both SASB and SACB strategies. As single assemblies are more numerous than coassemblies, there is thus a higher probability to reconstruct, independently, several times the same MAG. Finally, using this simulated data set, we did not detect any significant differences in the quality of MAGs reconstructed by the four strategies, neither in their completeness nor in their contamination levels (Fig. S7).

10.1128/msystems.00432-22.7 FIG S7 Comparison of the quality of MAGs reconstructed with each strategy on the mock data set. HQ, high quality; MQ, medium quality. (A) Completeness of HQ MAGs; (B) completeness of MQ MAGs; (C) contamination of HQ MAGs measured with CheckM; (D) contamination of MQ MAGs measured with CheckM; (E) contamination of HQ MAGs, measured with GUNC; (F) Contamination of MQ MAGs, measured with GUNC. Download FIG S7, PNG file, 0.1 MB. Copyright © 2022 Churcheward et al. 2022 Churcheward et al. https://creativecommons.org/licenses/by/4.0/ This content is distributed under the terms of the Creative Commons Attribution 4.0 International license.

### Comparing assembly-binning strategies on real metagenomes.

To further compare the four genome reconstruction strategies, we applied them to a real metagenomic data set, which is more complex in terms of species diversity and composition. Human gut microbiome studies represent a large fraction of publicly available metagenomes and are also good case studies as they represent metagenomes with intermediate complexity compared to soil or ocean metagenomes. Thus, we focused on analyzing a selection of 150 metagenomes of human gut microbiomes from the Integrative Human Microbiome Project (HMP) (40).

**FIG 4:**
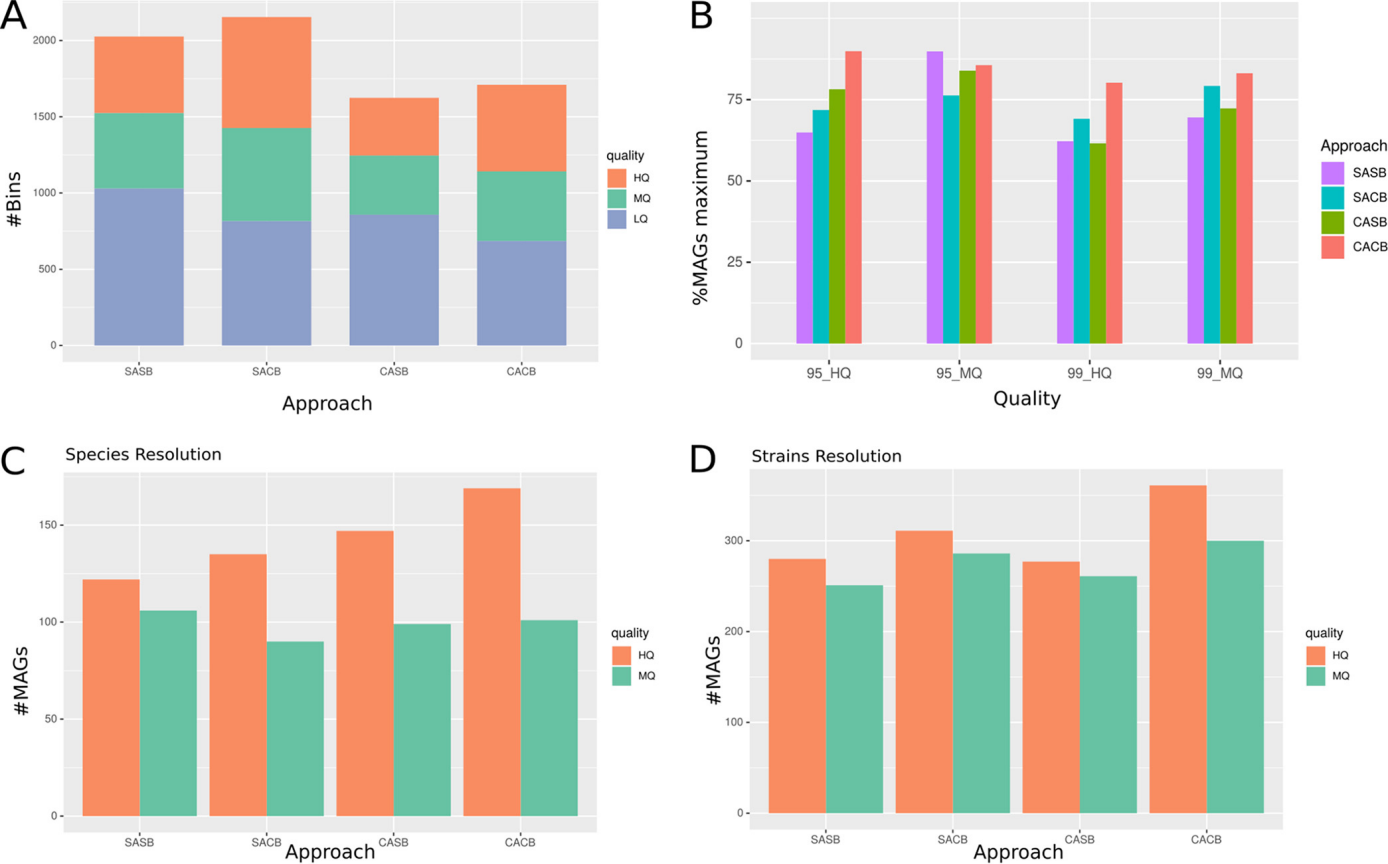
Evaluating the binning strategies on the HMP data set. (A) Total number of bins reconstructed per strategy. Colors represent the MAG qualities, estimated with CheckM. (B) Proportion of MAGs reconstructed for each strategy, after common dereplication of the four strategies, at the species resolution (95% identity) or at the strain resolution (99% identity). Number of dereplicated MAGs from each strategy is compared to the number of maximum expected MAGs, which is the number of MAGs obtained after dereplication of all the four strategies together. (C, D): Number of reconstructed MAGs after independent dereplication using dRep for each binning strategy, at (C) species resolution, consisting of a 95% ANI score dereplication; and (D) strain resolution, consisting of a 99% ANI score dereplication. SASB, single-assembly single binning; SACB, single-assembly cobinning; CASB, coassembly single binning; CACB, coassembly cobinning; HQ, high quality; MQ, medium quality; LQ, low quality.

Here, the MD-based clustering approach identified 64 metagenomic clusters to coassemble. When comparing all four strategies before dereplication, both single-assembly strategies reconstructed more genome bins than both coassembly strategies (Fig. 4A). Next, in order to determine how many MAGs we could expect to reconstruct at best by each strategy, we dereplicated altogether genome bins reconstructed by all strategies. The resulting number of dereplicated MAGs thus represents the highest number of MAGs we would be able to reconstruct with the HMP data set combining all four strategies. We then compared each strategy by considering what proportion of the maximum number of MAGs each strategy was able to reconstruct (Fig. 4B). After dereplication at the species level, despite the fact that single-assembly strategies recovered more bins, we observed that both coassembly strategies reconstructed more MAGs than single-assembly strategies. Also, for both coassembly and single-assembly strategies, the cobinning actually allowed to reconstruct more MAGs than the single-binning approach (Fig. 4B and C), which underlines the importance of integrating cross-sample information when binning genomes. However, after dereplication at strain level, we observed that the SACB strategy reconstructed more MAGs than CASB, while the SASB strategy reconstructed more HQ MAGs than the CASB strategy (Fig. 4B and D).

We also compared the MAGs quality (completeness and contamination) produced by each assembly-binning strategy. Differences in completeness were only observed between the SACB and CASB strategies, with SACB HQ MAGs being more complete than CASB HQ MAGs (Fig. S8A). Here, we also used both checkM (SCG-based) and GUNC (taxonomy-based) complementary approaches to estimate contamination. GUNC was able to detect more subtle differences in contamination between strategies than the checkM algorithm (Fig. S8). These observed differences demonstrated that cobinning strategies actually produced less contaminated MAGs than single-binning strategies, at all MAGs quality levels. Overall, these distinct results when dereplicating MAGs at species or strain level suggest that no single strategy can fit all needs. Therefore, the choice of an assembly-binning strategy should be informed by a biological question and considering the microbiome complexity under study.

10.1128/msystems.00432-22.8 FIG S8 Comparison of the quality of MAGs reconstructed with each strategy on the HMP data set. HQ, high quality; MQ, medium quality. (A) Completeness estimated for HQ MAGs; (B) completeness estimated for MQ MAGs; (C) contamination measured using SCGs for HQ MAGs; (D) contamination measured using SCGs for MQ MAGs; (E) contamination measured using all genes detected in the sequences of HQ MAGs; (F) contamination measured using all genes detected in the sequences of MQ MAGs. Download FIG S8, PNG file, 0.2 MB. Copyright © 2022 Churcheward et al. 2022 Churcheward et al. https://creativecommons.org/licenses/by/4.0/ This content is distributed under the terms of the Creative Commons Attribution 4.0 International license.

### Comparing MAGNETO to similar metagenomics workflows.

Finally, we compared the performances of MAGNETO to metagenomic workflows dedicated to MAG reconstruction, namely, METAWRAP (22), ATLAS (24) and nf-core/mag (25). We chose these three tools as they use similar software to perform assembly and binning, namely, MEGAHIT (41) and MetaBAT2 (9). The comparison of the workflows was performed using the HMP data set. ATLAS is a workflow only permitting single assembly of metagenomes, but integrates a binning refinement module using DAStool (42), which constitutes a good opportunity to evaluate whether single assembly could perform better after binning refinement. METAWRAP also contains a binning refinement module, albeit less complex than the DAStool methodology. This refinement module performs pairwise alignment of MAGs to detect redundant genomes, to then only conserve MAGs showing the best quality among detected duplicated MAGs. nf-core/mag uses the exact same tools as our workflow to perform assembly and binning. As compared to ATLAS, we observed that MAGNETO systematically reconstructed more MAGs using any of the four assembly-binning strategies (Table 1). However, it reconstructed less MAGs than METAWRAP. The higher number of MAGs produced by METAWRAP may be explained by its refinement module coupling several binners, as these binners may reconstruct more nonredundant MAGs, thus increasing their numbers. However, MAGNETO and nf/core-mag reconstructed the same number of MAGs for both CASB or CACB strategies. These similar results are most likely explained by the absence of a bin refinement module and by the fact that in both workflows, the binning step used the exact same parameters.

**TABLE 1:** Number of reconstructed MAGs for the HMP data set using different workflows, and different strategies, after dereplication at strain level^*a*^

Pipeline	Strategy	MAGs	
		HQ	MQ
ATLAS	SASB	253	120
METAWRAP	SASB	302	295
	SACB	320	242
	CASB	377	320
	CACB	386	350
nf-core/mag	CASB	277	261
	CACB	361	300
MAGNETO	SASB	280	251
	SACB	311	286
	CASB	277	261
	CACB	361	300

a MAGs: number of dereplicated MAGs; HQ: High Quality (Completeness > 90%, Contamination < 5%), MQ: Medium Quality (Completeness > 50%, Contamination < 10%).

### Design and implementation.

**FIG 5:**
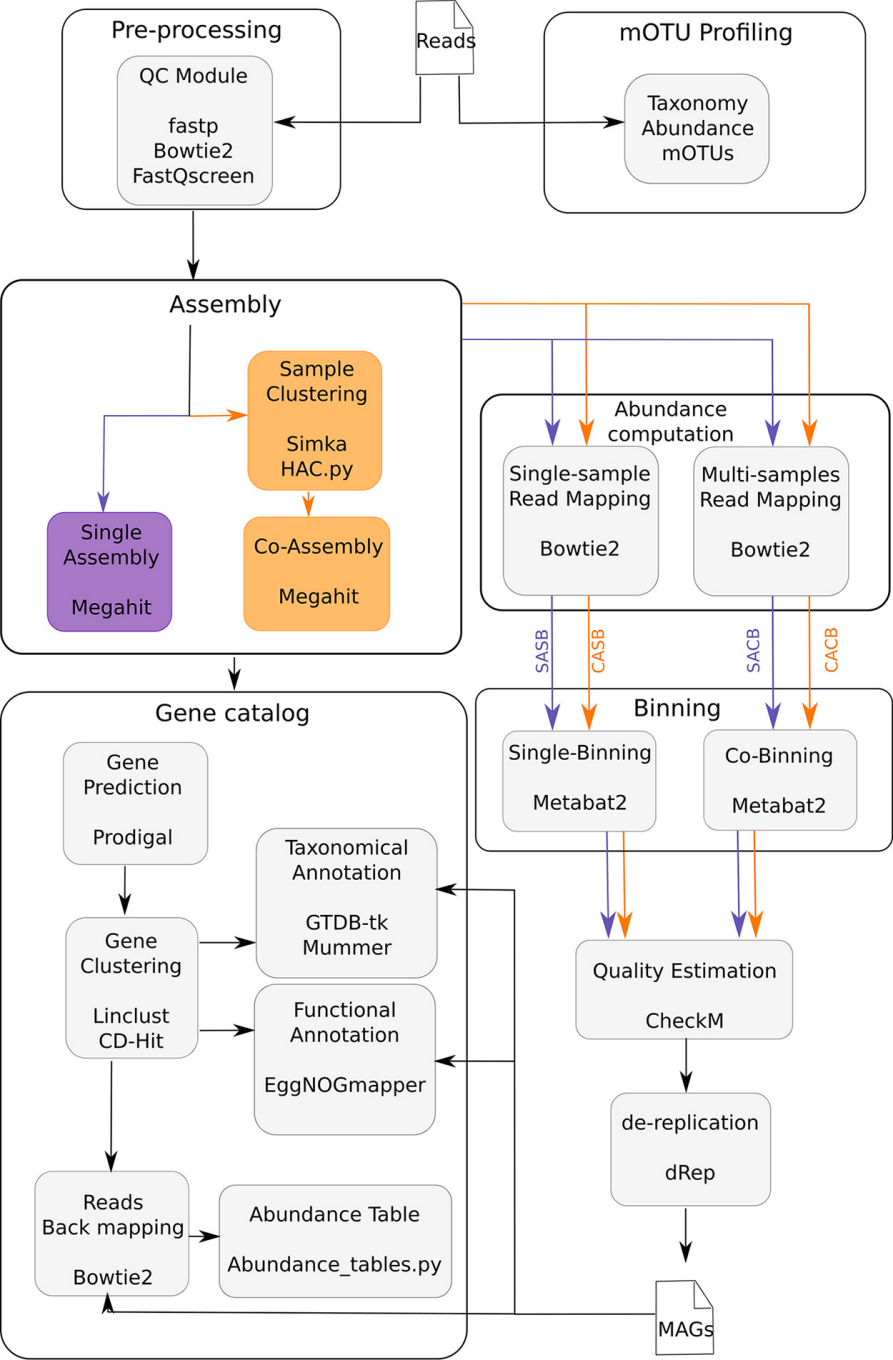
Overview of the MAGNETO workflow summary view of modules implemented in the MAGNETO workflow, with the name of the software or script associated with each task. The workflow can be launched for a complete run, to process raw reads into a gene catalog and MAGs, but each module can also be run independently. In purple: path to perform single assembly, corresponding to SASB (single assembly single binning) and SACB (single assembly cobinning) strategies, and orange: path to perform coassembly, corresponding to CASB (coassembly single binning) and CACB (coassembly cobinning) strategies.

MAGNETO is a Snakemake (43) workflow connecting open-source bioinformatics software, all available from Bioconda and conda-forge. Snakemake was chosen for its flexibility, its capacity to run both locally and on clusters, and its Conda management automating software installation. MAGNETO includes several tools designed for metagenomic studies. First, reads trimming is performed using fastp (44) and FastQ Screen (45). The coassembly module relies on Simka (35), which estimates metagenomic distances between samples based on their k-mers composition. MEGAHIT (41) then performs reads assembly/coassembly. We use MetaBAT2 (9) to bin contigs, and we assess the quality of bins using CheckM (46). The dereplication of bins into MAGs (bins of at least high of medium quality) is performed using dRep (37). Notably, MAGNETO can also be used to establish gene catalogs, to better capture metagenomic gene diversity by producing a nonredundant set of genes through sequence clustering at a user-defined sequence identity cutoff (e.g., 95%) using Linclust (47). GTDB-tk (48) is used to perform taxonomic annotation of dereplicated MAGs, and eggNOG-mapper (49) is used to perform the functional annotation of MAGs as well as the gene catalog (see Fig. 5).

A more complete description of each module implemented in MAGNETO is available in the Material and Methods section. The four binning strategies are directly configurable by the user, and a quick configuration allows performing from a single to all strategies for reconstructing MAGs. Notably, MAGNETO is currently the unique workflow providing an automated approach to define clusters of metagenomes for coassembly. Importantly, MAGNETO and nf-core/mag are also the only workflows allowing users to perform a cobinning strategy. A synthetic comparison of functionalities provided by the workflows tested in this study is available in Table 2.

**TABLE 2:** Comparison of tasks performed by evaluated workflows

Steps	ATLAS	METAWRAP	nf-core/mag	MAGNETO
Preprocessing				
Reads trimming	✓	✓	✓	✓
Contamination	✓	✓	✓	✓
Assembly				
Coassembly possible		✓	✓	✓
Compute sets to coassemble			✓	
Binning				
Cobinning possible		✓	✓	✓
Multiple binning software	✓	✓		
Bin refinement	✓	✓		
Bin reassembly	✓	✓		
Postprocessing				
MAGs quality check	✓	✓	✓	✓
Dereplication step	✓	✓	✓	✓
Genome annotation	✓	✓	✓	✓
Gene catalogue			✓	✓
Reproducibility				
Workflow management	✓		✓	✓
Packages management	✓		✓	✓

## DISCUSSION

In this work, we present MAGNETO, a fully automated workflow enabling genome-resolved metagenomics. It implements a novel approach to compute clusters of metagenomes for coassembly without *a priori* knowledge, as well as complementary assembly-binning strategies to maximize MAG recovery toward specific goals. MAGNETO also provides key functionalities, from the construction and annotation of gene catalogs to the generation of genes and genomes abundance matrices.

### An unsupervised approach to metagenomic coassembly.

We demonstrated the utility of a nonsupervised metagenomic-distance based approach to guide metagenomics coassembly on a large set of ocean metagenomes. Indeed, clusters of metagenomes identified by the MD-based approach did not overlap with oceanic regions previously used for guiding coassembly of these metagenomes (13). As anticipated, this implies that, in the ocean, geographic distances do not necessarily reflect compositional metagenomic distances between microbial communities. This observation can likely be explained by the fact that the composition of marine microbial communities is significantly structured through environmental filtering by key abiotic factors such as temperature (28) and ocean currents influencing species dispersal (50). Interestingly, the MD-based clustering analysis grouped together in a single cluster (cluster #1; see Fig. 1A) of metagenomes from sampling stations facing upwelling currents. As upwelling regions are influenced by deep ocean currents raising cold nutrient-rich waters to the surface, they can significantly impact species diversity of marine microbial communities toward richer states (51, 52).

The rationale behind our metagenomic distance-based approach to perform coassembly was to infer which metagenomes should be grouped together in an unsupervised fashion without *a priori* knowledge. The aim was to develop an approach that could guarantee the actual closeness of the metagenomes to coassemble, thus emphasizing the increase in species-specific reads abundance for the assembler. Although the coassembly of closely related metagenomes has been shown to erode contigs quality (12, 15), we could show that our approach did not increase fragmentation or misassemblies within contigs (Fig. S4). In fact, our MD approach reconstructed MAGs that are more complete and less contaminated than the OR approach (Fig. S4). Although both metrics we used to estimate MAG contamination reported contradictory results, we argue that GUNC (38) likely provides better estimates of contamination as it is based on a much larger set of genes compared to CheckM (46), which assess contamination solely based on SCGs. As SCGs represent highly-conserved genes across all taxa, coassembling similar metagenomes may actually increase the probability of assembling or binning core regions of closely related genomes. A higher fragmentation of the genomes was already observed following the coassembly of metagenomes with closely related strains (15, 53), although it was also shown not to affect completeness or the contamination of coassembled genomes (37). Accessory regions may thus be less affected by coassembly, although they are also generally more difficult to bin (12).

We observed a very high number of exclusive MAGs between the OR and MD approaches, namely, 525 for MD and 575 for OR, representing 31.2% and 33.3% of the MAGs reconstructed by each approach, respectively (Fig. 1B). This result indicates that, even if our approach performs better in terms of reconstructed MAG quality, it nevertheless does not capture the same information from metagenomes compared to the OR approach. This is confirmed by the increase in proportion of recruited reads when back-mapping to combined MAGs from both approaches (Fig. 1C). Thus, combining the MD approach with a coassembly based on *a priori* knowledge (when available) may represent a good opportunity to better capture the actual bacterial diversity in metagenomes. However, the proportion of mapped reads was significantly higher on MD MAGs compared to OR MAGs when considering only detected MAGs in samples (Fig. 1D). Here, we could show that the OR approach reconstructed MAGs recruiting a higher proportion of reads, but that this higher proportion was mainly driven by MAGs displaying lower horizontal coverage (<30%), suggesting these MAGs contained relatively small genomic regions recruiting a high proportion of reads. These observations, coupled with the smaller contamination observed in OR MAGs when estimated using SCGs, may imply that the OR approach allows a better reconstruction of core genomic regions, which are shared among a higher proportion of organisms.

Applying the MD-based coassembly approach on the HMP data set, we found that the identified clusters of metagenomes mostly corresponded to the IBD pathology affecting the patients (Fig. S9). Indeed, a majority of clusters containing metagenomes from healthy patients did not contain any metagenomes related to IBD (16 out of 23 clusters contained non-IBD metagenomes), and a majority of the clusters containing CD or UC patients are composed of metagenomes associated with only the same type of IBD (26 out of 34 clusters contained IBD metagenomes). This observation emphasizes the relevance of our method, as changes in the composition of the gut microbiota have been associated with IBD diagnostic (40, 54, 55).

10.1128/msystems.00432-22.9 FIG S9 Composition of the MD clusters obtained with the HMP data set. For each cluster, the number of metagenomes is shown, with the IBD diagnosis associated with each metagenome. Diagnoses: non-IBD, healthy; CD, Crohn’s disease; UC, ulcerative colitis. Download FIG S9, PNG file, 0.1 MB. Copyright © 2022 Churcheward et al. 2022 Churcheward et al. https://creativecommons.org/licenses/by/4.0/ This content is distributed under the terms of the Creative Commons Attribution 4.0 International license.

### A systematic comparison of assembly-binning strategies.

When comparing the four different assembly-binning strategies we defined herein, we observed that costrategies systematically reconstructed more MAGs than single strategies. Notably, the CACB strategy was identified as the best performing in terms of number of recovered MAGs, across all (simulated and real) data sets we considered. This may be explained by (i) the increase in (rare) reads abundance through the coassembly, and (ii) the higher amount of coabundance information integrated into the cobinning process (7, 26). On simulated data sets, coassembly strategies systematically reconstructed more MAGs after dereplication, while applying single-binning or cobinning. However, this was not the case when analyzing the HMP data set, for which the SACB strategy reconstructed more strain-level MAGs than CASB. This may be due to an uneven distribution of strains across metagenomes. Indeed, human gut microbiomes tend to be personal and usually exhibit higher inter- than intraindividual community variations at strain level (56, 57). Overall, if gut strains are individual-specific and thus only occur in a low number of metagenomes, coassembly will be less effective to actually increase strain-specific reads for improving their assembly. This result suggests that an MD-based approach integrating single-nucleotide polymorphism (SNP) information would be useful to improve the reconstruction of strain-level MAGs.

### A multisample assembly-binning strategy maximizes genomes recovery.

We showed that coassembly approaches usually reconstructed higher numbers of (MQ) MAGs, albeit with a tendency to be more contaminated (HQ MAGs). As previously reported (12), this underlines the utility of coassembly to recover rare or less-abundant genomes, and to maximize MAGs recovery from a limited number of metagenomes. Here, cobinning strategies (SACB and CACB) systematically reconstructed less contaminated MAGs than single-binning strategies (SASB and CASB) in data sets for which differences in MAG quality could be detected between strategies. Thus, multisample coabundance information computed across a minimum number of metagenomes appears particularly relevant to improve genome binning and to limit the erroneous grouping of contigs. However, the cobinning strategy may represent a severe limitation as it requires larger computational resources (CPU time and disk space), since it implies performing *N*^2^ reads mapping operations, with *N* the total number of metagenomes. For the CAMI data set, differences in MQ MAG quality between strategies were in contradiction with analyses of the other data sets, although the HQ MAG comparison pointed toward similar conclusions as in the other data sets. This may be explained by the different number of MAGs reconstructed between each strategy. The 80 MAGs reconstructed by the SASB strategy may belong to abundant organisms, thus implying a smaller risk of increased contamination. However, as SACB and CASB reconstructed almost twice the number of MAGs compared to SASB, the MQ MAGs recovered by these strategies may belong to less abundant genomes; hence, these MAGs may be harder to reconstruct with a few samples (*n* = 5) and thus may be more prone to contamination.

Interestingly, the effect of the coassembly step on MAG contamination is unclear. So far, only a few methods, including CheckM and GUNC, exist to estimate MAG quality. When considering CheckM on the HMP data set, single-assembly strategies reconstructed less contaminated MAGs than coassembly strategies. However, when considering contamination estimated by GUNC, coassembly strategies constructed less contaminated MAGs. These results underline the crucial need to develop more accurate methods to properly estimate MAG quality, and also highlight the utility of using complementary strategies to estimate genome quality.

Coassembly constitutes a useful and affordable strategy for shallow sequenced metagenomes or when the number of metagenomes to coassemble is limited. In such cases, the increase in complexity of the assembly is limited, thus removing the main computational limitation of coassembly. Similar to coassembly, cobinning is also affected by metagenomic sequencing depth, as the computation time obviously increases with the number of reads. As demonstrated, the cobinning strategy represents a powerful and useful, although computer-intensive, strategy when numerous samples are available, as it helps to reconstruct more HQ MAGs. A potential perspective for improving the cobinning process would be to identify an optimal number of samples to compute coabundances in order to optimize its cost–benefit ratio.

## MATERIALS AND METHODS

### Reads preprocessing.

Raw reads were filtered using fastp (44) and FastQ Screen (45). fastp filters reads on the quality, length. and complexity. FastQ Screen is a tool allowing control of contamination within metagenomic samples, by mapping their reads to reference genomes. These two tools provide results reports to the user that are useful to evaluate the quality of reads.

### Assembly.

We performed reads assembly using MEGAHIT (41), as this assembler provides an excellent trade-off between computational requirements and assembly quality (58). metaSPAdes (59) could have been considered as it provides better performances than MEGAHIT in terms of overall percentage of the metagenome recruited in the assembly (60) and maximum length of scaffolds produced (60, 61). However, this performance increase occurs at the cost of a greater consumption of computational resources (58) and a presence of a greater proportion of misassembled sequences in contigs than MEGAHIT (60). More importantly, metaSPAdes was originally not designed to perform coassembly, which constituted a major drawback in our workflow. Moreover, MEGAHIT is able to capture microdiversity from the metagenomes more efficiently than metaSPAdes, as it discards less low-abundant reads during assembly (58). Coassemblies of the marine metagenomes were performed using the *–presets meta-large* option, as these metagenomes revealed to be highly complex. All other assemblies were performed using the *–preset meta-sensitive* option.

### Coassembly strategy.

In order to determine which samples to coassemble, we used Simka, a *de novo* and scalable tool for comparative metagenomics (35). Simka computes different distances based on *k*-mer counts, instead of species counts. In our case, we used their modified Jaccard (or *AB*-Jaccard) distance rather than the default Bray-Curtis distance, as the latter does not satisfy triangle inequality. Once the distance matrix from Simka was computed, samples were then clustered using a Ward-based hierarchical agglomerative clustering (62). Then, we iteratively cut the dendrogram and assessed partitioning quality using the Silhouette method (36).

### Genome binning strategies.

Binning was performed using MetaBAT2 (9), as it is currently one of the fastest and best performing genome binners. We set the minimum length for contigs to be binned to 1500 nucleotides. As MetaBAT2 uses composition and abundance to perform binning, a preliminary step to map reads back to assembled contigs was performed to measure abundance. Reads mapping was achieved using Bowtie2 (63).

Instead of computing an abundance metric only from the metagenome assembled into contigs, MetaBAT2 may compute a coabundance metric using contig coverage from several samples, even if these samples do not participate in the assembly. A coabundance metric computed from several samples increases the quality of the genome bins produced (64). Depending on the number of samples used to compute contigs abundance, the corresponding metric is either an abundance or a coabundance metric. Thus, two strategies can be pursued in order to perform binning: (i) single binning, which uses abundance of contigs measured from assembled metagenome(s); or (ii) cobinning, which uses coabundance of contigs measured from all the metagenomes of a data set. Combined with the decision to perform either single assembly or coassembly, we defined four binning strategies: single assembly of one metagenome with single binning (SASB), single assembly of one metagenome with cobinning (SACB), coassembly of one set of metagenomes with single binning (CASB), and coassembly of one set of metagenomes with cobinning (CACB).

### Genome bins quality.

Genome bin quality was defined by two metrics, namely, completeness and contamination. Completeness measures the fraction of the initial genome captured, while completeness measures the fraction of alien sequences; both rely on the presence–absence patterns of universal single-copy marker genes (SCGs). To assess genome bins quality, we used CheckM (46) and GUNC (38). Based on contamination and completeness, we distinguished three standard quality levels for bins (65): (i) high-quality (HQ) bins with completeness >90% and contamination <5%, (ii) medium-quality (MQ) bins with completeness >50% and contamination <10%, while the (iii) low-quality (LQ) bins are bins that are neither HQ nor. Only HQ and MQ bins were then considered to be MAGs. The comparisons of reconstructed MAGs quality from different strategies were performed using Mann-Whitney U test using R (66). As a MAG may be reconstructed independently in either two (or more) samples or two (or more) cosamples, MAGs are also dereplicated using dRep (37). Two MAGs were considered to be duplicated if their pairwise ANI (average nucleotide identity) score was above a given identity threshold *t* (*t* being a percentage of sequence identity) on more than 60% of their bases (67). We considered two different values for *t*: *t *= 0.95, which corresponds to a dereplication at species level, and *t *= 0.99, which corresponds to a dereplication at strain level (68).

### Genome annotation module.

Functional and taxonomic annotations were performed for the strain-level MAGs collection, which encompasses the species-level collection. To perform functional annotation of MAGs, we used eggNOG-mapper (49), and we used GTDB-tk (48) to perform taxonomic annotation. Finally, reads of each sample were mapped back onto both species- and strain-level MAG collections using Bowtie2, and an abundance table was produced using an in-house Python script.

### Gene annotation module.

Coding DNA sequences (CDSs) were detected on assembled contigs per sample (single assembly) using Prodigal (69). Genes from all samples were clustered at 95% identity using Linclust (47) in order to produce a nonredundant set of genes (gene95 collection). EggNOG (49) and MMSEQ2 (70) were used to annotate this gene collection, for functional and taxonomic information, respectively. Finally, reads from each sample were mapped back onto the gene95 collection using Bowtie2, and an abundance table was produced.

### Data sets.

The marine metagenomes data set corresponds to the same 93 oceanic metagenomes as processed in Delmont et al. (13), which are available at the European Bioinformatics Institute (EBI) repository under project ID ERP001736. In order to benchmark the assembly-binning strategies, we simulated two different mock metagenome data sets. First, we used the CAMI (15) high-complexity data set, which is a 75-Gbp time series data set sequenced into short reads, composed of five samples from a high-complexity community with correlated log normal abundance distributions (596 genomes and 478 circular elements). However, the data set above did not allow us to assess our clustering method, which was not able to determine an optimal clustering to perform CASB and CACB. Thus, a simple coassembly strategy, gathering all 5 samples, was performed on this data set and was referred as a CASB strategy. We also simulated two more data sets (Text S1; available at https://doi.org/10.5281/zenodo.6613455), composed of more samples than the CAMI data set, in order to better evaluate our coassembly module. The first data set we simulated was composed of 10 metagenomes containing different abundances of 20 genomes, with each metagenome of size 1 Gbp. Our second data set was composed of 20 metagenomes containing different abundances of 100 genomes, each sample of size1 Gbp. Both these simulated data sets were generated using CAMISIM (39) with each metagenome’s size fixed to 1 Gbp. Each metagenome contained the 100 source genomes, with specific abundance distribution created by sampling from a log-normal distribution with *μ* set to 1 and *σ* to 2 (default values). To simulate the first mock data set, we used the 20 test genomes used by default in CAMISIM. The 100 genomes used for the simulation of the second mock data set were randomly sampled from CAMI high-complexity source genomes. Other CAMISIM parameters were set to their default values.

For the human gut microbiome data set, we collected raw data from the Integrative Human Microbiome Project (iHMP) (40). Based on the availability of both metagenomics and metatranscriptomics data, we selected a subset of 150 metagenomes (Text S1; available at https://doi.org/10.5281/zenodo.6613455), representing 80 individuals and patients followed during 1 year. Metagenomes were extracted from stool samples and were sequenced using Illumina technology. The samples subset we analyzed here was composed of three sample groups of strictly equal size characterized by diagnosis, with individuals with no inflammatory bowel disease (non-IBD) and patients either diagnosed with Crohn’s disease (CD) or Ulcerative Colitis (UC).

10.1128/msystems.00432-22.1 TEXT S1 Supplemental Methods. Download Text S1, PDF file, 0.03 MB. Copyright © 2022 Churcheward et al. 2022 Churcheward et al. https://creativecommons.org/licenses/by/4.0/ This content is distributed under the terms of the Creative Commons Attribution 4.0 International license.
