# Supplementary figures and images for: MAGNETO: An Automated Workflow for Genome-Resolved Metagenomics
Source: mSystems. 2022 Jun 15;7(4):e00432-22. doi: 10.1128/msystems.00432-22 (PMC9426564; doi:10.1128/msystems.00432-22)

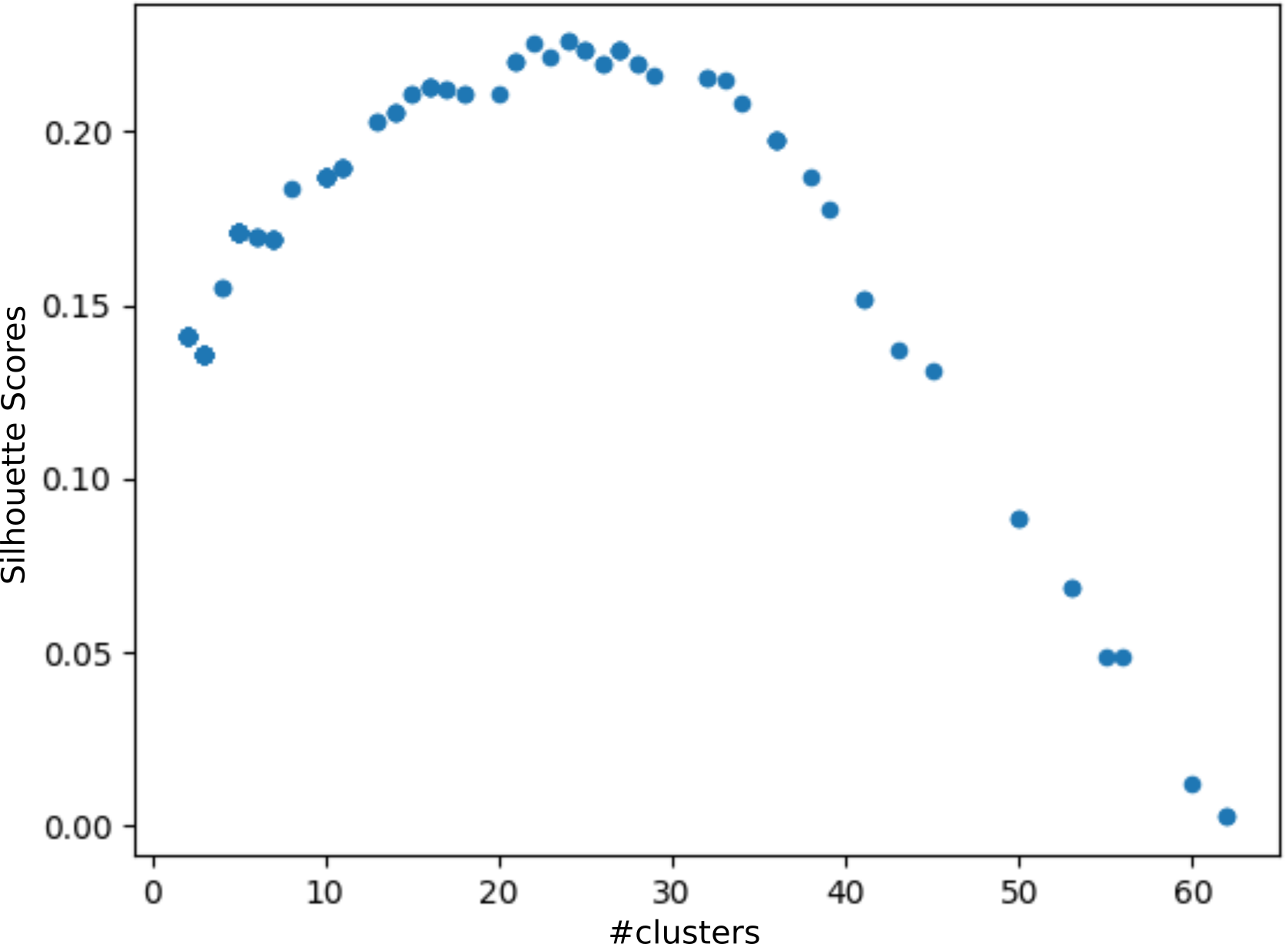

Supplement: FIG S1 [file msystems.00432-22-s0001.png]

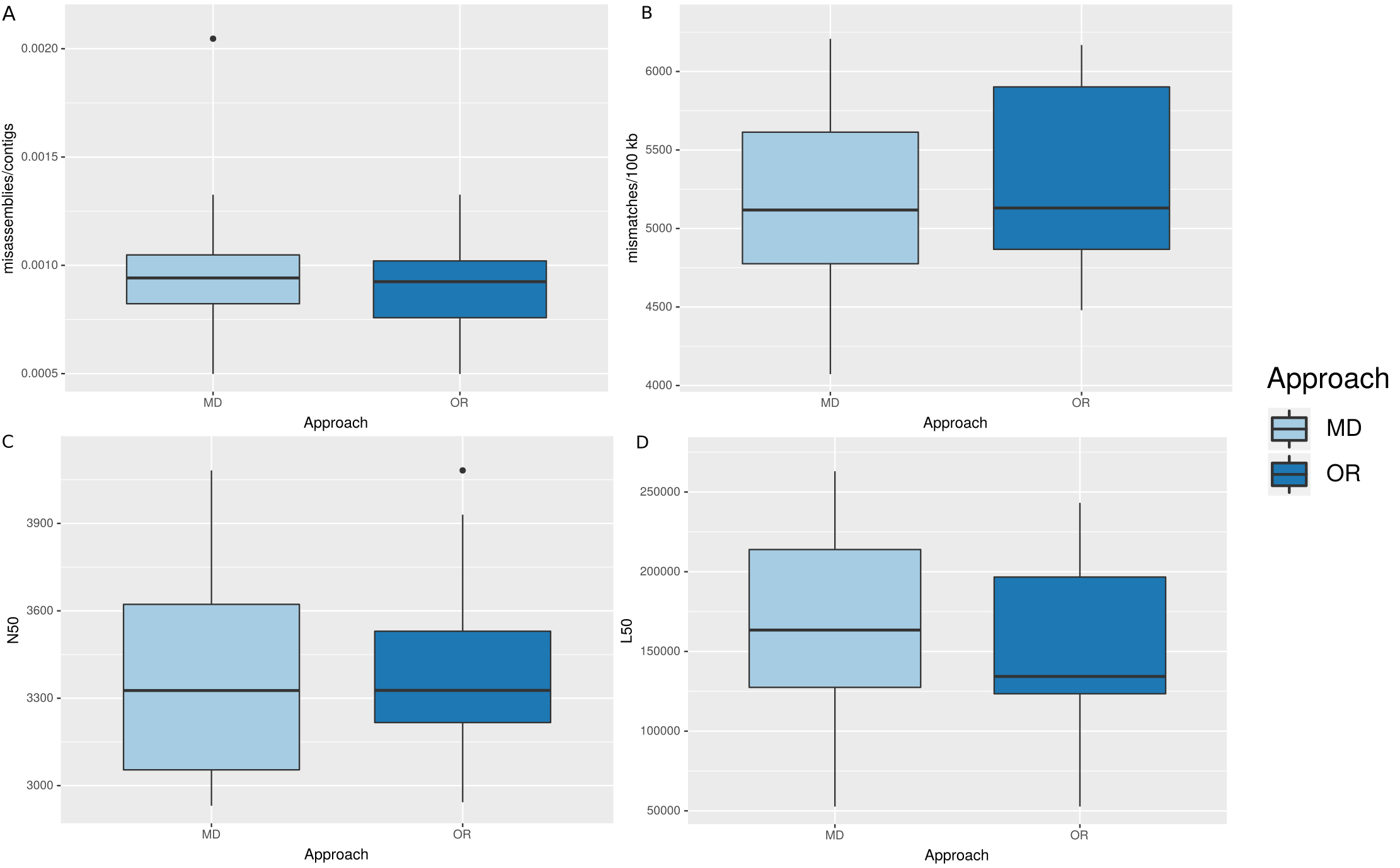

Supplement: FIG S2 [file msystems.00432-22-s0002.png]

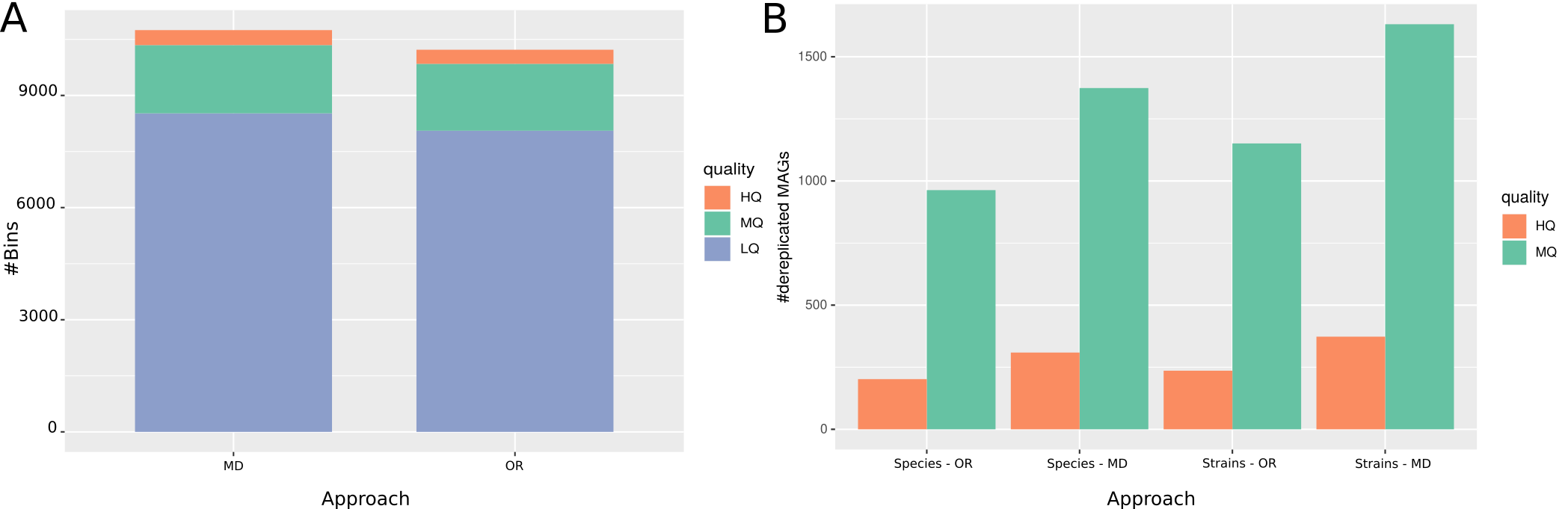

Supplement: FIG S3 [file msystems.00432-22-s0003.png]

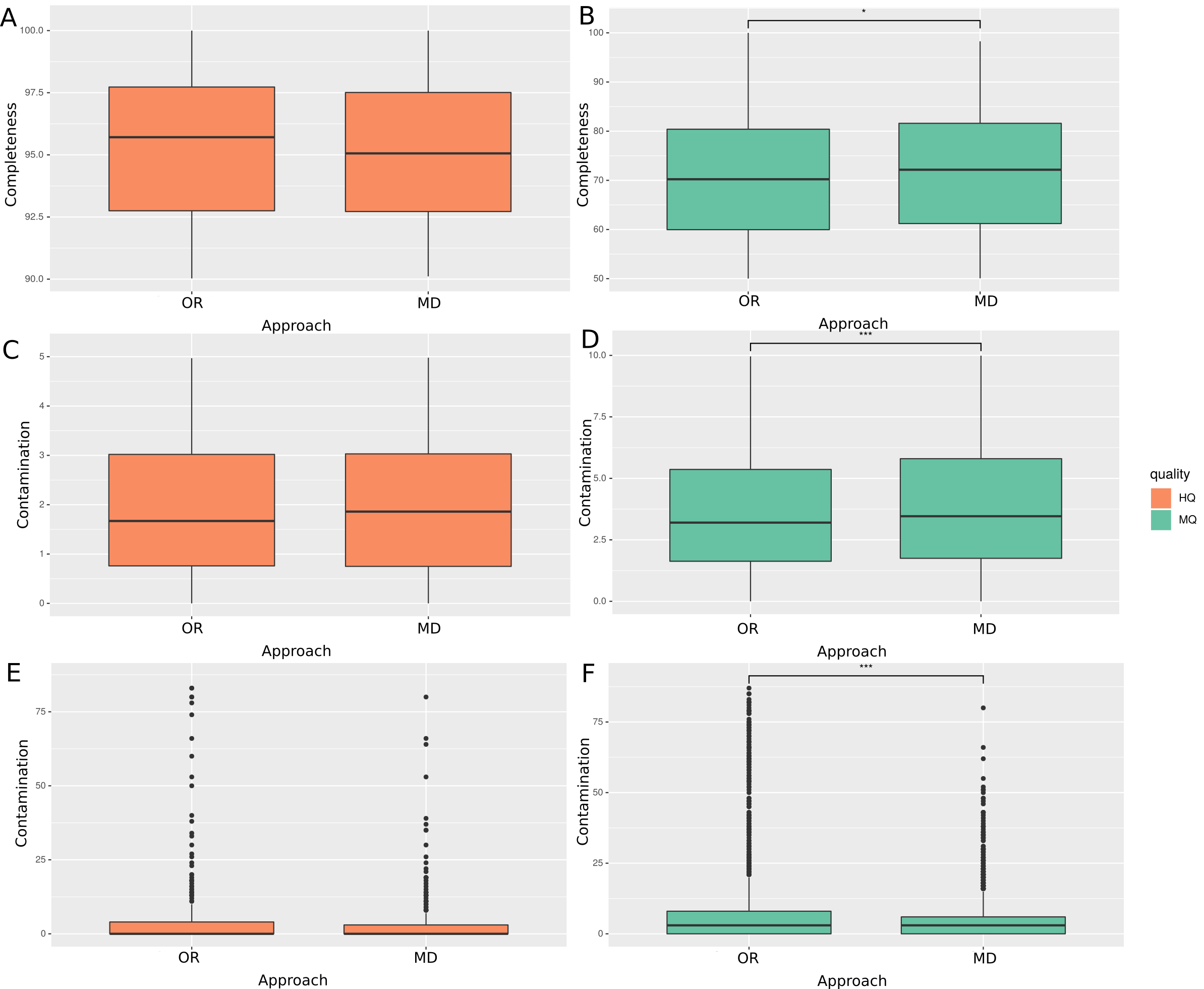

Supplement: FIG S4 [file msystems.00432-22-s0004.png]

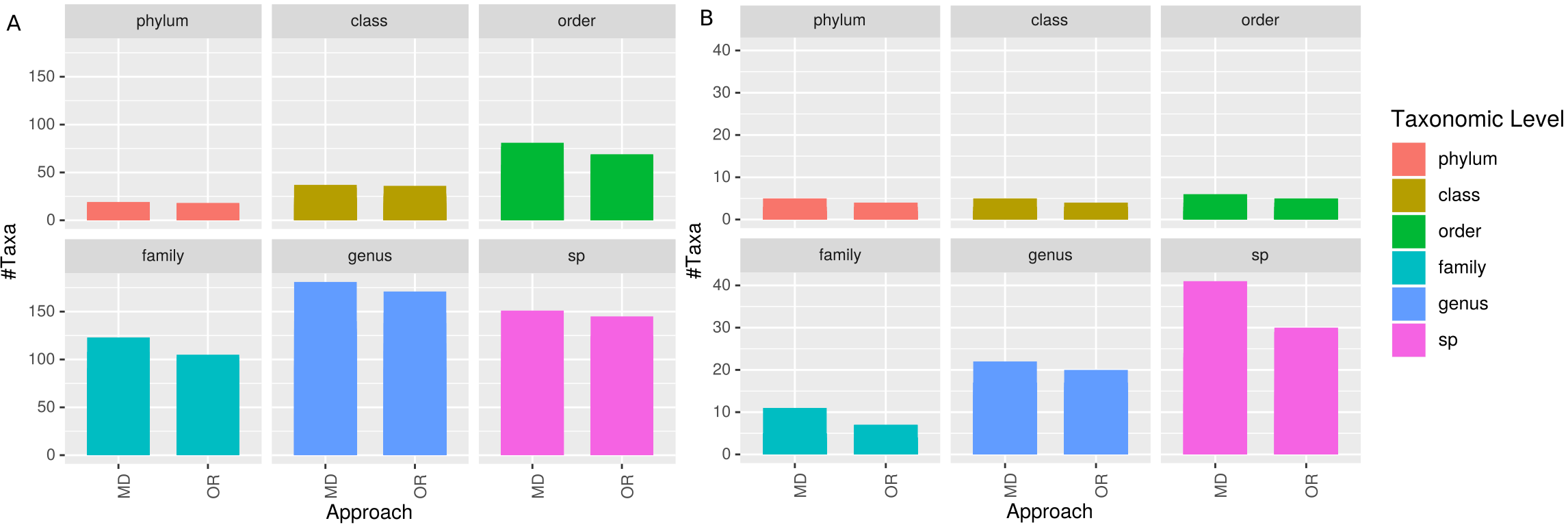

Supplement: FIG S5 [file msystems.00432-22-s0005.png]

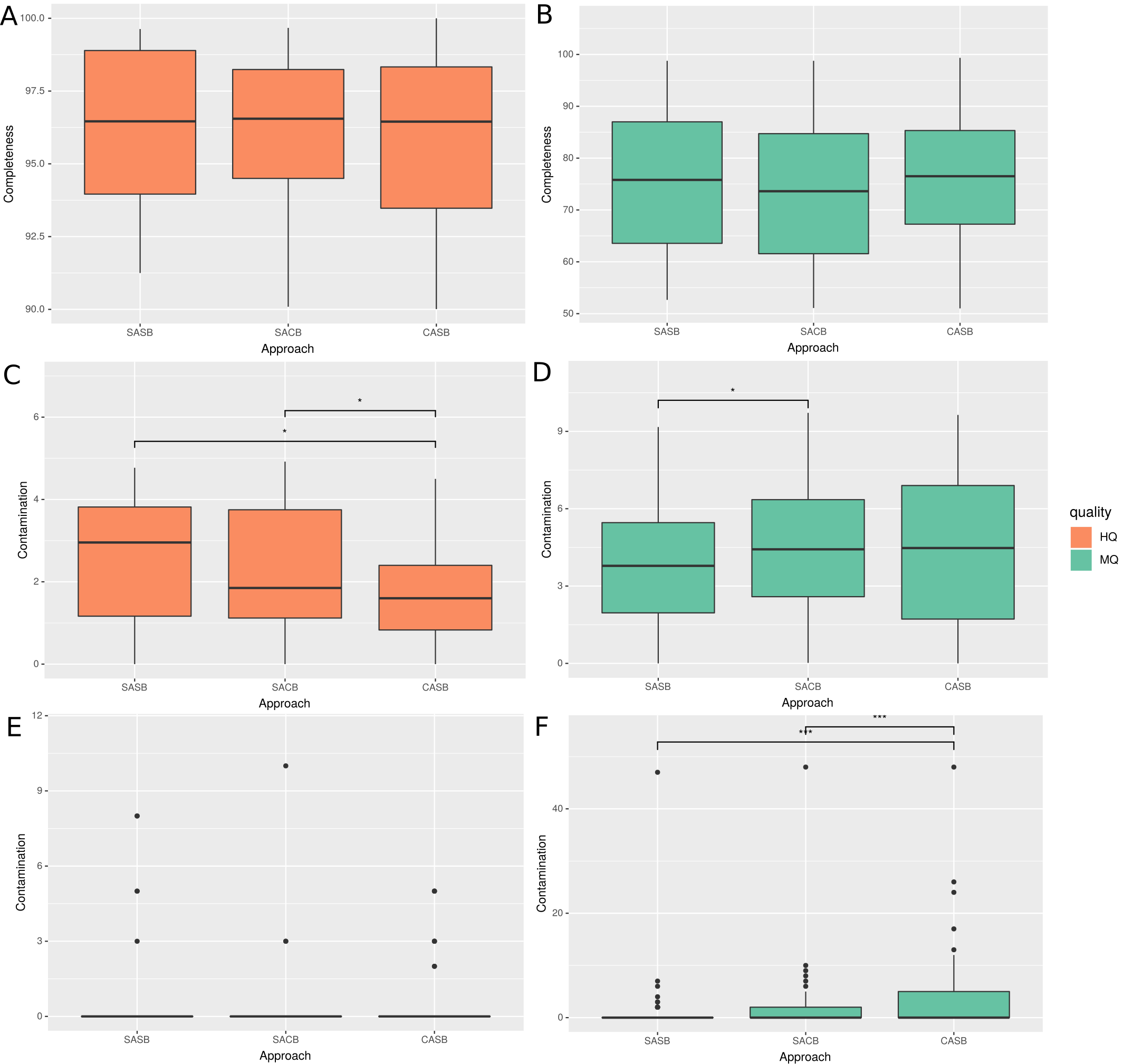

Supplement: FIG S6 [file msystems.00432-22-s0006.png]

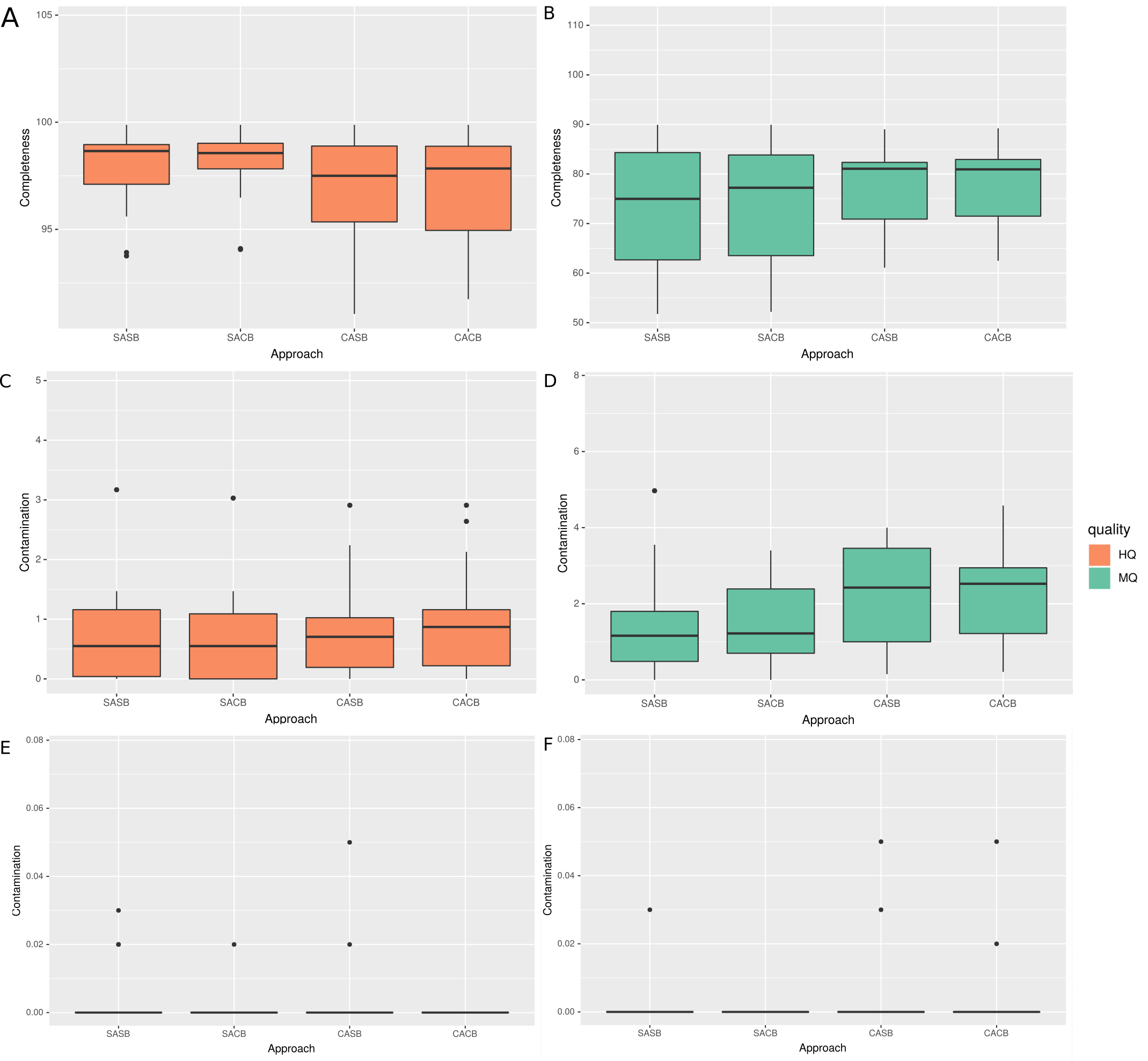

Supplement: FIG S7 [file msystems.00432-22-s0007.png]

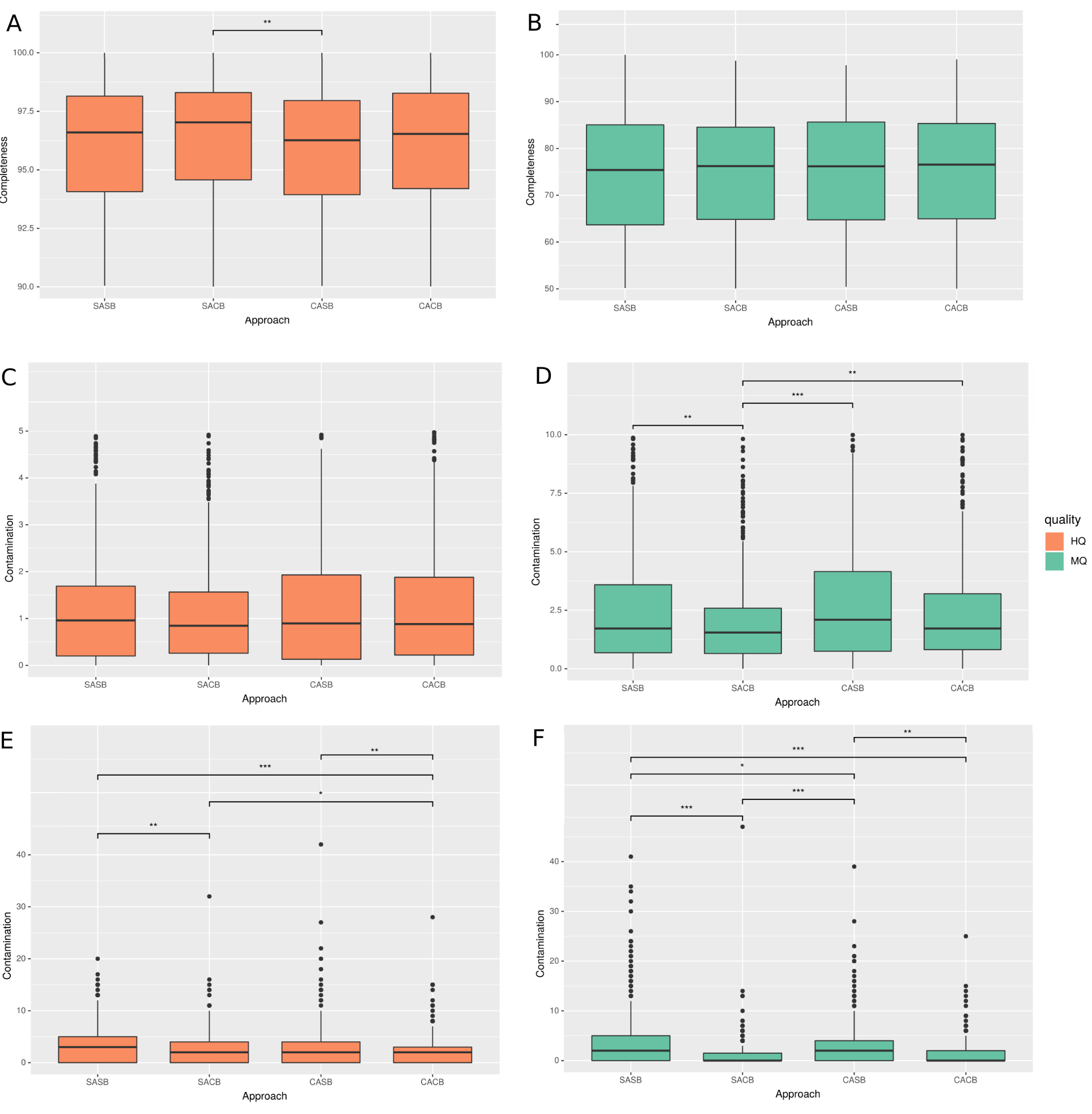

Supplement: FIG S8 [file msystems.00432-22-s0008.png]

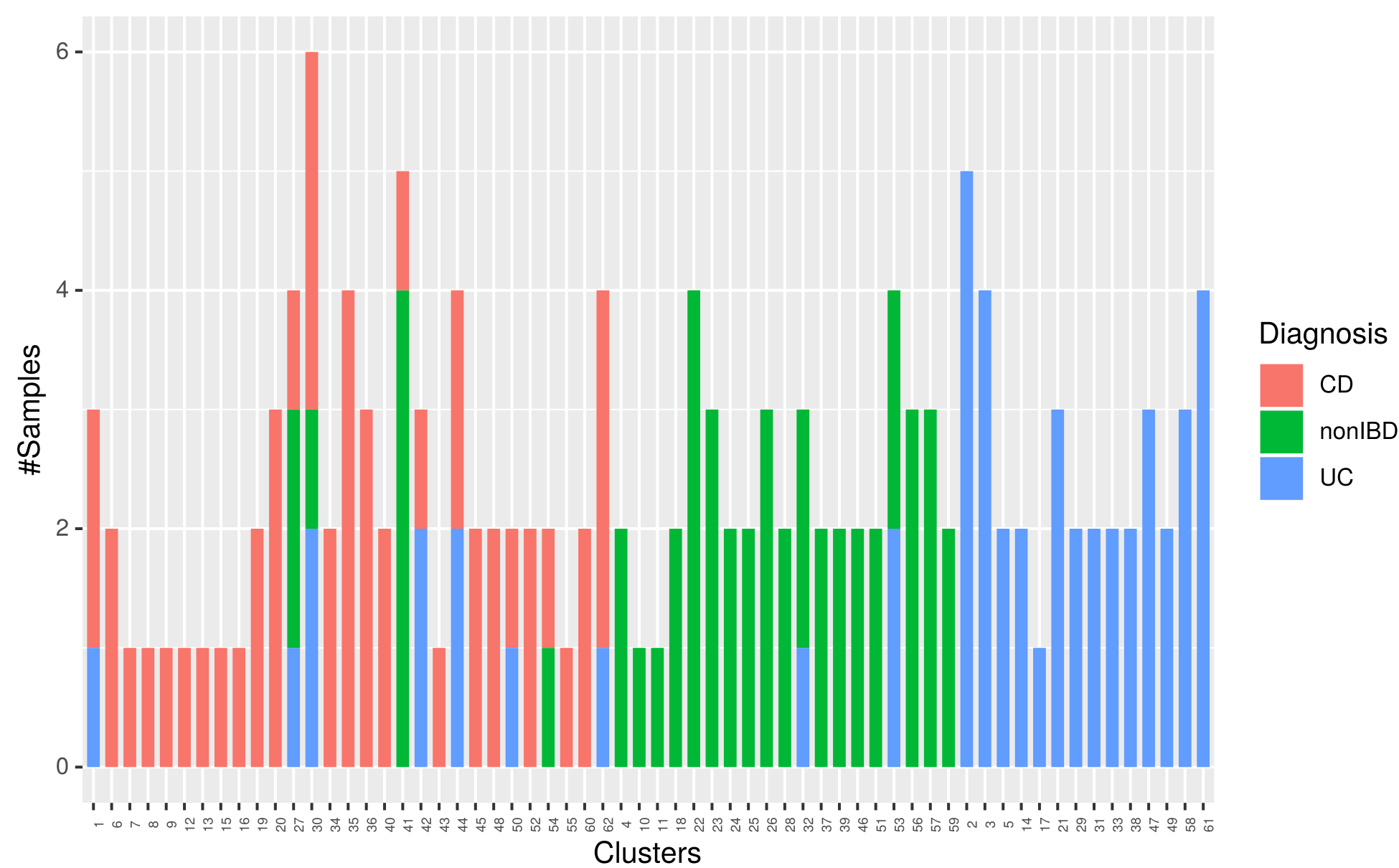

Supplement: FIG S9 [file msystems.00432-22-s0009.png]
