# Supplementary material for: MAGNETO: An Automated Workflow for Genome-Resolved Metagenomics
Source: mSystems. 2022 Jun 15;7(4):e00432-22. doi: 10.1128/msystems.00432-22 (PMC9426564; doi:10.1128/msystems.00432-22)
Supplement: TEXT S1 [file msystems.00432-22-s0010.pdf]

SUPPLEMENTARY MATERIAL

Supplementary Methods The construction of the Fig. 1B panel implied a global de-replication of the *TARA* Oceans MAGs, performed by combining sets of MAGs reconstructed with both approaches using dRep as described in the methods section. Once this global de-replication was performed, the total number of de-replicated MAGs (dMAGs) related to one approach is thus:

$N_{ir} = n_{ir} + m_{ijr}$

with n the number of dMAGs already reconstructed by the approach i_i, and m_{ij} the number of dMAGs reconstructed by approach j, but located in a de-replication cluster containing at least 1 MAG reconstructed by the approach i, at the de-replication resolution r. From the dRep output, we can identify the de-replication cluster each MAG belongs to, and the number of members located in the same de-replication cluster. We can then list, for a given de-replication resolution r, the set of dMAGs related to one approach i, searching for each non-unique dMAG (*i.e.* having at least 1 neighbour in its de-replication cluster) of the approach j, if there is at least one MAG from the approach i. Thus, to detect shared dMAGs between both approaches, we identified the common elements between the four sets of N_{ir} dMAGs.

To detect shared genomes between sets of genomes related to different de-replication resolutions, we should precise that the set of dMAGs at species-level for one approach is completely included in the set of dMAGs at strain-level from this same approach. Thus, we can non-ambiguously identify clustering relationships between two MAGs from different de-replication levels, or find exclusive MAGs reconstructed by one approach, but present at both species and strain levels.
